# Different Perception of Musical Stimuli in Patients with Monolateral and Bilateral Cochlear Implants

**DOI:** 10.1155/2014/876290

**Published:** 2014-08-07

**Authors:** Anton Giulio Maglione, Giovanni Vecchiato, Carlo Antonio Leone, Rosa Grassia, Franco Mosca, Alfredo Colosimo, Paolo Malerba, Fabio Babiloni

**Affiliations:** ^1^Department of Anatomy, Histology, Forensic Medicine and Orthopedics, University Sapienza, Piazzale Aldo Moro 5, 00185 Rome, Italy; ^2^Department of Physiology and Pharmacology, University Sapienza, Piazzale Aldo Moro 5, 00185 Rome, Italy; ^3^ENT Department, Azienda Ospedaliera Dei Colli Monaldi, 80131 Naples, Italy; ^4^Cochlear Italia Srl., Via Larga 33, 40138 Bologna, Italy; ^5^BrainSigns Srl, via Sesto Celere 7/c, 00152 Rome, Italy

## Abstract

The aim of the present study is to measure the perceived pleasantness during the observation of a musical video clip in a group of cochlear implanted adult patients when compared to a group of normal hearing subjects. This comparison was performed by using the imbalance of the EEG power spectra in alpha band over frontal areas as a metric for the perceived pleasantness. Subjects were asked to watch a musical video clip in three different experimental conditions: with the original audio included (Norm), with a distorted version of the audio (Dist), and without the audio (Mute). The frontal EEG imbalance between the estimated power spectra for the left and right prefrontal areas has been calculated to investigate the differences among the two populations. Results suggested that the perceived pleasantness of the musical video clip in the normal hearing population and in the bilateral cochlear implanted populations has similar range of variation across the different stimulations (Norm, Dist, and Mute), when compared to the range of variation of video clip's pleasantness for the monolateral cochlear implanted population. A similarity exists in the trends of the perceived pleasantness across the different experimental conditions in the mono- and bilaterally cochlear implanted patients.

## 1. Introduction

Most cochlear implanted users report difficulties with music perception even after many years of implant usage [1, 2]. This seems to be due to the fact that the signal processing of the cochlear device provides only limited spectral information and produces a much narrower dynamic range than acoustic hearing [3]. Limitations in the appreciation of the timbre and pitch of musical tones sequences have been reported in cochlear implant patients when compared with normal hearing subjects [4]. One of the main problems related to the perception of music is the assessment of the intrinsic pleasure derived from the related listening. The self-reported psychological scales are often inadequate to convey precise information about the cerebral processing related to the pleasantness of the perceived music. However, in these last years, objective measures of the cerebral activity have been put in strict relation to the pleasantness of an individual experienced perception [5]. In fact, indirect variables of emotional processing could be gathered by tracking variations of the activity of specific anatomical structures linked to the emotional processing activity in humans, such as the pre- and frontal cortex (PFC and FC, resp.) [6–8]. The PFC region is structurally and functionally heterogeneous, but its role in the generation of the emotions is well recognized. EEG spectral power analyses indicate that the anterior cerebral hemispheres are differentially lateralized for approach and withdrawal motivational tendencies and emotions. Specifically, findings suggest that the left PFC is an important brain area in a widespread circuit mediating appetitive approach, while the right PFC appears to form a major component of a neural circuit that instantiates defensive withdrawal [9].

The main goal of the present study is to apply the estimation of EEG rhythms to measure and to investigate differences in the perceived pleasantness experienced by monolateral and bilateral cochlear implanted adults during the observation of a musical video clip (MCI and BCI groups, resp.). The bilateral group was composed of the same monolateral implanted patients that participated to the first EEG recordings. They were enrolled in the bilateral group after surgically received their second cochlear implant. The subjects of the BCI group were ready for the second EEG recordings only once the audiometric tests returned a good setup of the second implant. This usually happens between three to five months after the second implant. In order to quantify the amount of perceived pleasantness, a frontal EEG spectral imbalance has been estimated in agreement with the available literature [6, 7, 9]. The experimental stimuli were realized by the generation of three different versions of the same video clip: the first one was with the original audio included (Norm condition) and the second one was characterized by the reversed audio track (Dist), while the third one is without any music (Mute). The gathered EEG data set has been compared in terms of frontal spectral imbalance within these three experimental conditions. In addition, an age-matched control group formed by normal hearing adults was also recorded under the same experimental conditions (e.g., Norm, Distort, and Mute) for the comparisons with the patients groups.

## 2. Materials and Methods

### 2.1. Subjects

A group of seven patients having a monolateral cochlear implant (MCI) was selected for similar characteristic of their pathology as subjects for this study. All the patients gave their informed consent to the study that was approved by the ethical committee of the institutions involved in the experimentation. In addition, all the patients would like to receive a second cochlear implant. In the month before EEG registration, all patients had their monolateral cochlear implant (Cochlear Ltd.) controlled and mapped with a mixed behavioral and objective method and received a warble-tone free-field audiometry and a comprehensive speech perception and recognition assessment. All of patients had all CI electrodes active with normal impedance levels and were using an ACE strategy, a 900 pps stimulation rate, and an ADRO preprocessing algorithm. On the day, the registration was performed; patients received a warble-tone free-field audiometry and a speech audiometry to make sure their hearing and speech recognition abilities were good. Some days after the first EEG recordings, the same group of patients was successively undergone to a surgical operation to install a second cochlear implant, again from Cochlear Ltd. Such group of patients (the same patients of MCI) is named successively in the analysis bilateral cochlear implant (BCI) group. The group (CTRL) is composed of seven aged-matched adults with no history of hearing diseases.

### 2.2. Experimental Design and EEG Data Recording

The visual stimuli consisted of a four-minute-length piece from the musical West Side Story. The musical was chosen given the significant impact of music in such video clip. In fact, in the video clip there are no words but only dance and music. Three versions of the video clip were viewed by each subject of the different groups: (i) the original video plus the original music (Norm); (ii) the original video with a distorted and unpleasant version of the music (Dist), and the original video with no sound associated with it (Mute condition). The Dist condition was obtained by reversing the audio stream of the original video clip, in order to leave the global acoustic pressure generated on the patients and normal hearing controls during the experiment unchanged. The stimulus order of the Norm, Dist, and Mute conditions was randomized between all clips in order to eliminate the “Sequence” factor as possible conditioning effect in the successive analysis.

### 2.3. EEG Recordings

For the electroencephalographic recording (EEG), the subjects were comfortably seated in a chair in a dimly lit room. A 16-channel EEG system (BeMicro, EBNeuro spa, Italy) was used with the following electrode positions: AF7, AF8, F3, Fz, F4, T7, T8,C3, Cz, C4, T8, P3, Pz, P4, O1, and O2. The EEG data were acquired with impedance below 5 KΩ.

Before each EEG recordings related to the different video clip presented, 1 minute of EEG brain activity with open eye without stimulation was recorded, in order to be used as the reference condition in the successive analysis. At the end of each particular video stimulus presented, the patients were asked to give an explicit pleasantness score ranging from 1 (unpleasant) to 10 (maximally pleasant). A brief interview with the patient closed the experimental session.

**Figure 1:**
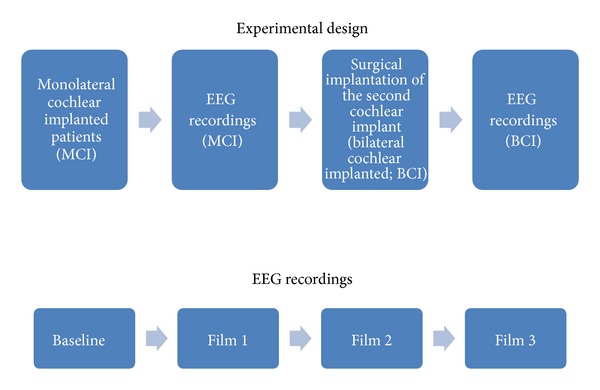
In the upper part of the figure the experimental design in which the patients with the monolateral cochlear implant (MCI) were first subjected to the EEG recordings and then submitted to a surgical procedure to insert the second cochlear implant is presented. After several months from the surgery, when the patients recovered a good hearing condition, a second EEG recording session was made. In the lower part of the figure there is the sequence characterizing the EEG recordings performed in each population (MCI, BCI). Films 1, 2, and 3 were randomly assigned to the Norm, Dist, and Mute film, to avoid bias due to the sequence of the videos presentation.

The general experimental design is presented in Figure 1.

After the acquisition, EEG data were subjected to the Independent Component Analysis in order to remove the eye-blink artifacts, in agreement with the methodologies presented elsewhere [10]. On the artifact free EEG data, the power spectral density (PSD) with the Welch method was then estimated in the alpha frequency band. Such particular frequency band was defined not by using a predetermined frequency limits (such as 8–12 Hz) for all the subjects but rather by using the individual alpha frequency (IAF) determination according to the previous published literature [11–23]. In particular, the alpha band was defined within the limits [IAF-4, IAF+2] where the IAF is the individual alpha frequency [24].

### 2.4. Estimation of the Imbalance Spectral Index

According to the EEG frontal asymmetry theory the imbalance index has been defined such that positive values are related to left alpha desynchronization. Thus, positive values of this index indicate the perception of pleasant stimuli and vice versa for the unpleasant stimuli. The EEG power spectral imbalance in the alpha band has been calculated for all the recorded subjects during the three experimental conditions according to the following formula:

(1) Imbalance  Index  =log⁡(|PSDR−PSDL|)∗sign⁡(PSDR−PSDL),

where PSD_R_ and PSD_L_ indicate the power spectral density calculated in the alpha band over the frontal electrodes, respectively, on left and right hemisphere, log is the logarithm on base 10, and sign⁡(·) is the sign function. Such newly defined index is linked to the previous definition of the imbalance activity of the prefrontal cortices as provided by the work of Davidson [7, 9], but it is here adopted by taking the logarithm of such imbalance. This choice has been performed since in the comparison between different subjects the logarithm transformation decreases the variances of the gathered sample. This property is particularly appropriate in this context as it is characterized by a small sample size [25]. The logarithmic transformation then leaves the properties of the statistical tests successively performed on such data unchanged.

According to the work of Davidson [9], the frontal imbalance index is then used as a measure of the pleasantness generated in a subject, during the course of an experimental task.

### 2.5. Statistical Analysis

The value of the estimated spectral imbalance index was first standardized when compared to the baseline condition occurring during the eye-open condition. The *z*-scores obtained were then compared across the three experimental groups (CTRL, MCI, and BCI). The Analysis of Variance was performed by using the factor Movie, related to the different video stimulations provided with the video clip, with 3 levels (Norm, Dist, and Mute). Such ANOVAs were performed to assess if there were variations of the imbalance index for the CTRL, MCI, and BCI populations. Duncan post hoc tests were performed with a level of 5% of significance.

## 3. Results

**Figure 2:**
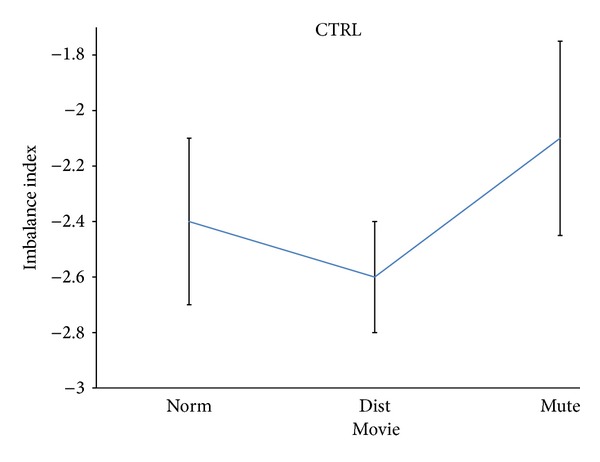
Mean and standard deviation of the normalized imbalance indexes across the different experimental conditions of the factor Movie (Norm, Dist, and Mute) for the population of normal hearing subjects (CTRL). Post hoc test reveal a statistical significant difference across the different levels of Movie factor at the 5% significance level.

The ANOVAs returned a significant impact of the factor Movie for all the populations involved in the study. In particular, the factor Movie resulted in statistical significant with *P* < 0.05 for the CTRL group, while it was significant with *P* < 0.003 for the MCI group and with a level of *P* < 0.05 for the BCI group.  Figure 2 presents the mean value of the imbalance index in the age-matched CTRL population. It must be noted that for the *z*-score imbalance index, the zero level is relative to the absence of particular positive or negative attitude when compared to the baseline condition. The standard deviation bars are also presented around each average value for the CTRL population investigated. On the *x*-axes, the three different conditions for the Movie factor are represented, for example, Norm, Dist, and Mute. In the CTRL population the level of the imbalance index was in general negative while the Mute condition was slightly preferred on average when compared to the other kind of stimulations (e.g., Norm and Dist). It must be noted that the general values of the average levels of the imbalance index were quite similar across the different stimulations (between −2.1 and −2.6) with an excursion of 0.5 normalized unities.

Duncan's post hoc tests revealed significant differences across the different levels of the Movie factor at the 5% significance level. In particular, the Dist condition was judged the worst by the normal hearing population, when compared to the others.

**Figure 3:**
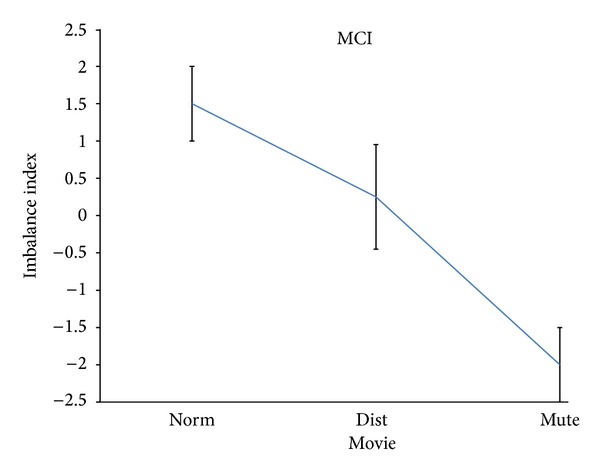
The spectral imbalance index for the monolateral cochlear implanted (MCI) population. Same conventions as in Figure 1.

Figure 3 showed the results of the ANOVA performed on the data related to the MCI population across the different values of the factor Movie (e.g., Norm, Dist, and Mute).

In this case the worst perceived condition by the MCI population was that related to the Mute condition, being the excursion quite large across the different experimental conditions observed. In fact, values ranged from 1.5 on average for the Norm Movie condition to the −2.0 value for the Mute condition, with an total excursion of the pleasantness index of about 3.5 normalized units. All the Duncan's tests revealed statistical significant differences between the different imbalance indexes in the examined conditions, at the 5% statistical significance.

**Figure 4:**
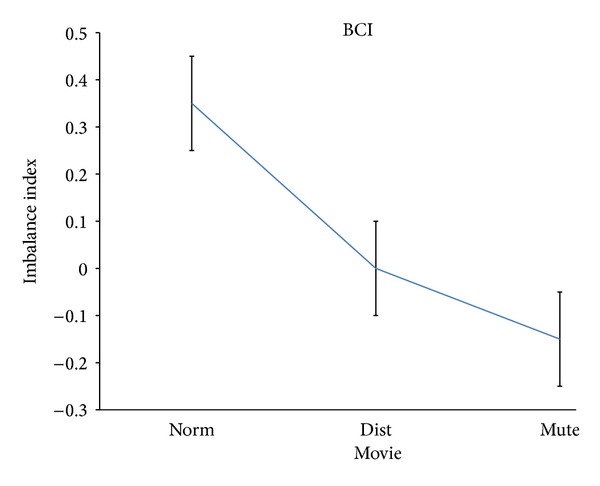
The spectral imbalance index for the bilateral cochlear implanted (BCI) population. Same conventions as in Figure 2.

Figure 4 presents the average values of the imbalance index for the BCI population. It is worth noticing that the BCI group was composed by the same patients of the MCI group, once they received the implant of the second cochlear device. It can be appreciated that the excursion of the imbalance index estimated for the BCI group was quite small, starting from an average value of 0.4 *z*-score units for the pleasantness during the Norm stimulation and moving down to −0.15 for the Mute condition, with a total excursion of 0.55 *z*-score units.

**Figure 5:**
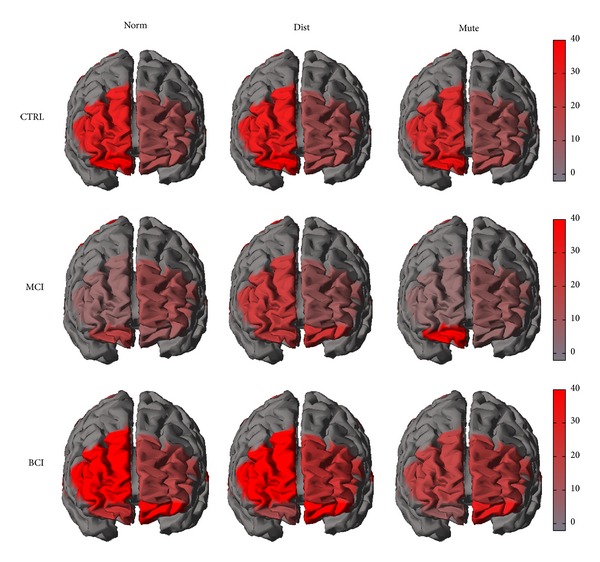
The brain is seen from a frontal perspective and the increase or decrease of EEG power spectra in the alpha band is presented for all the conditions (Norm, Dist, and Mute) and the groups investigated (CTRL, MCI, and BCI).

Also in this case the Duncan test reveals a statistical significant difference between the examined conditions at the 5% level. A representation of the cortical activity that is estimated from the acquired data is presented in Figure 5. The brain is seen from a frontal perspective and the increase or decrease of EEG power spectra in the alpha band is presented for all the conditions (Norm, Dist, and Mute) and the groups investigated (CTRL, MCI, and BCI).

## 4. Discussion

In the present study, an approach to the estimation of the perceived pleasantness during the listening of music in groups of cochlear implanted adults and normal hearing subjects has been presented by using the EEG imbalance index. In fact, such index correlates with the perceived pleasantness in humans according to the literature available [7–9]. The patients of this study are a group of monolateral cochlear implanted adults (MCI) that performs a first EEG recording with the Norm, Dist and Mute experimental conditions described above. After such EEG recordings, all the patients of MCI group undergone to a surgery to receive the second cochlear implant. After the second cochlear implant was properly tested by clinicians and the patients were adapted to them, such group of patients (now the BCI group) was subjected to the second EEG recordings with the same stimulations. This study presents a couple of methodological issues that need to be discussed before to analyze the results obtained. First, the sample size adopted in the present study appears to be quite moderate. In fact, it may be argued that seven patients for each considered group (MCI and BCI) are not enough to draw robust conclusions from a statistical point of view. However, it could be remembered that all the patients of the MCI group after being recorded undergone surgical implantation of the second cochlear device. Thus, the intersubject variance is greatly reduced by considering the same patients in the MCI and BCI groups, increasing the hypothesized statistical power of the conclusions here reported. A second issue is related to the fact that there is an unavoidable bias of the experimental conditions, since every element of the BCI group has already watched the proposed experimental setup when he/she participated in the MCI group. As anticipated, such bias cannot be avoided unless a different population of bilateral cochlear implanted patients would be recruited. However, in this case a larger number of patients would be necessary for both the MCI and BCI groups to compensate the intersubjects intrinsic variability of this kind of measurements. It can be stated then that the presented results indicate possible trends that have to be confirmed with a study using a larger sample size.

It is stated that the results of this study suggested a significant variation of the pleasantness's perception of the musical video clip across the different tested experimental conditions (Norm, Dist, and Mute) in the analyzed populations (CTRL, MCI, and BCI). However, this variation is less pronounced in CTRL and BCI group than in the MCI group. In fact, while the excursions of the average values of the imbalance index for CTRL and BCI groups were quite similar across the experimental conditions (around 0.5 of the imbalance index normalized units, see Figures 2 and 4), the excursion of the average values for the MCI group was remarkably higher (around 3.4 of the imbalance index normalized units). Thus, it appears that after the second cochlear implant, the music perception and the related pleasantness became more uniform across the different stimulations provided, as it happens in the normal hearing subjects analyzed (CTRL). It is worth noticing that the imbalance index was negative in the control group for all the conditions provided. This describes a general situation of dislike for all the stimulations provided when compared to the baseline condition (e.g., eyes closed). Of particular interest is the fact that for the analyzed patients in both groups (MCI or BCI), the Mute condition was perceived as the worst among all the conditions. This particular result could be interpreted according to the idea that the worst condition for a patient that struggled with severe unilateral or bilateral hearing problems is the absence of sound. This interpretation was also corroborated by what has emerged from the interviews conducted to the patients. In fact, the patients stated that they remember the feeling of being deaf when they saw the mute video clips.

In summary, the presented results suggested the capability of the employed EEG techniques to measure differences in the perceived pleasantness of the music of groups of monolateral and bilateral cochlear implanted patients, with respect to an age-matched control group. As previously stressed, limitations of this study are related to the moderate sample size adopted, while the strengths are instead related to the fact that the same patients were analyzed before (MCI) and after (BCI) the implant of a second cochlear device.
